# 415. Epidemiological Evaluation of Antibiotic Resistance and Subsequent Effect on Healthcare Resource Utilization among Subjects with *Pseudomonas aeruginosa* Infections in Italy

**DOI:** 10.1093/ofid/ofac492.492

**Published:** 2022-12-15

**Authors:** Matteo Bassetti, Antonio Cascio, Annamaria Cattelan, Roberto Cauda, Francesco Menichetti, Nicola Petrosillo, Carolina Rescigno, Carlo Tascini, Antonio Vena, Pierluigi Viale

**Affiliations:** Department of Health Science (DISSAL), Infectious Diseases Unit,, Genova, Liguria, Italy; 1. U.O.C. Infectious and Tropical Diseases Unit- Department of Health Promotion, Mother and Child Care, Internal Medicine and Medical Specialties "G D'Alessandro," University of Palermo, Palermo, Italy, Palermo, Sicilia, Italy; Azienda Ospedaliera Padoa, Italy, Padova, Veneto, Italy; Dipartimento di Scienze di Laboratorio e Infettivologiche, Fondazione Policlinico Universitario A Gemelli IRCCS, Rome, Italy, Rome, Lazio, Italy; Department of Clinical and Experimental Medicine, Infectious Diseases Unit, University of Pisa, Pisa, Italy, Pisa, Toscana, Italy; Department of Clinical Research, National Institute for Infectious Diseases Lazzaro Spallanzani, Rome, Italy, Rome, Lazio, Italy; First Division of Infectious Diseases, Cotugno Hospital, AORN dei Colli, Naples, Italy, Napoli, Campania, Italy; Infectious Diseases Division, Department of Medicine, University of Udine, Azienda Sanitaria Universitaria Friuli Centrale (ASUFC), Udine, Italy, Udine, Friuli-Venezia Giulia, Italy; Infectious Diseases Unit, San Martino Policlinico Hospital, IRCCS for Oncology and Neurosciences, Genoa, Italy; Department of Health Sciences (DISSAL), University of Genoa, Genoa, Italy, Genova, Liguria, Italy; Infectious Diseases Unit, Department of Medical and Surgical Sciences, Policlinico Sant'Orsola Malpighi, University of Bologna, Bologna, Italy, Bologna, Emilia-Romagna, Italy

## Abstract

**Background:**

The aims of the study were to 1) evaluate the prevalence of multidrug resistant (MDR) /Extensively drug resistant (XDR) strains among hospitalized adults with *Pseudomonas aeruginosa* (*PA*) infections, and 2) examine whether antimicrobial resistance in *PA* infections is associated with worsening functional status and higher health care resource utilization (HCRU).

**Methods:**

This multicenter prospective study was conducted in 9 large Italian teaching hospitals between June 2018-February 2020. We included patients aged ≥18 years with a diagnosis of nosocomial pneumonia (NP), complicated urinary tract infections (cUTI) or complicated intra-abdominal infections (cIAI) due to *PA* as confirmed by local evaluation of microbiological results. MDR *PA* was defined as acquired non-susceptibility to at least one agent in three or more antimicrobial categories. XDR *PA* was defined as acquired non-susceptibility to at least one agent in all but two or fewer antimicrobial categories. HCRU metrics evaluated included hospital length of stay (LOS) and intensive care unit (ICU) LOS.

**Results:**

A total of 95 patients with a nosocomial infection due to *PA* were enrolled. The main baseline characteristics of overall patients were reported in **Table 1.** Almost one-third of patients (28.4%) reported either MDR or XDR *PA* infection, with more patients experiencing MDR **(Table 1).** Health care resource use stratified by patients with and without MDR/XDR status are reported in Table 2. Overall, in our study population, median hospital LOS and ICU LOS were 42.0 (IQR=39.0) and 15.5 (IQR=37.0) days, respectively. There was a statistically significant longer median hospital LOS for patients with MDR/XDR infections compared to non MDR/XDR PA infections (53.0 vs. 36.5 days, p=0.04). ICU LOS also trended towards being longer for patients with MDR/XDR infections compared to those with non-MDR/XDR infections (25.5 vs. 13.5 days, p=0.10).

**Figure:**
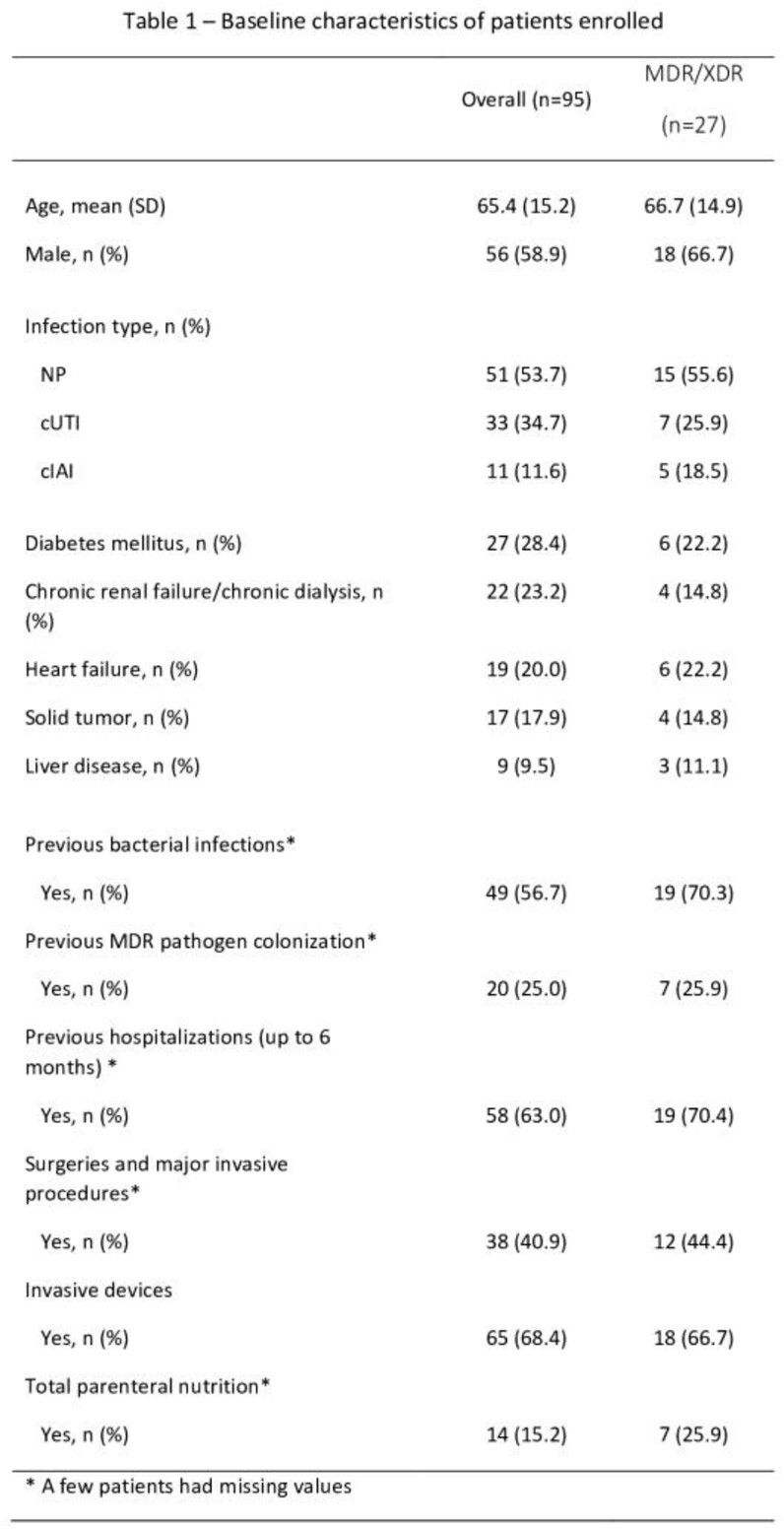

**Figure:**
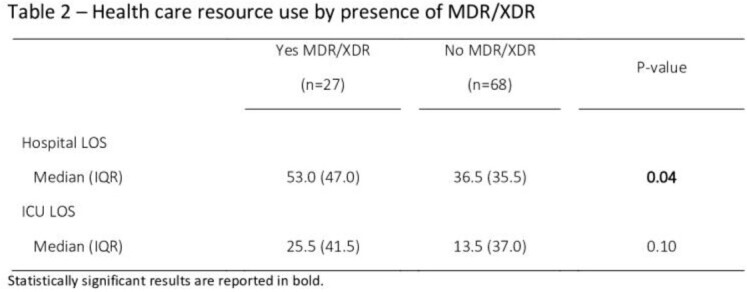

**Conclusion:**

MDR/XDR isolates were prevalent among patients with nosocomial infections due to *PA,* particularly in those with cIAI. Overall, the present study may suggest a positive correlation between having MDR-XDR *PA* nosocomial infections (NP, cUTI, and cIAI) and increased HCRU that require further attention from a disease management perspective.

**Disclosures:**

**Matteo Bassetti, PhD**, Angelini: Advisor/Consultant|Astellas: Grant/Research Support|Bayer: Advisor/Consultant|Bayer: Honoraria|BioMe ´ rieux: Advisor/Consultant|BioMe ´ rieux: Honoraria|Cidara: Advisor/Consultant|Cidara: Honoraria|Cipla: Advisor/Consultant|Cipla: Honoraria|Gilead: Advisor/Consultant|Gilead: Honoraria|Menarini: Advisor/Consultant|Menarini: Honoraria|MSD: Advisor/Consultant|MSD: Honoraria|Nabriva: Advisor/Consultant|Pfizer: Advisor/Consultant|Pfizer: Board Member|Pfizer: Grant/Research Support|Pfizer: Honoraria|Shionogi: Advisor/Consultant|Shionogi: Honoraria|Tetraphase: Advisor/Consultant **Francesco Menichetti, n/a**, Aneglini: Advisor/Consultant|Aneglini: Board Member|Aneglini: Grant/Research Support|Aneglini: Honoraria|Astellas: Advisor/Consultant|Astellas: Honoraria|Becton: Advisor/Consultant|Becton: Honoraria|bioMérieux: Advisor/Consultant|bioMérieux: Honoraria|Biotest: Advisor/Consultant|Biotest: Board Member|Biotest: Honoraria|Bristol-Myers Squibb: Advisor/Consultant|Bristol-Myers Squibb: Honoraria|Correvio: Advisor/Consultant|Correvio: Speaker honoraria|Dickinson: Advisor/Consultant|Dickinson: Honoraria|Gilead: Advisor/Consultant|Gilead: Grant/Research Support|Janssen: Advisor/Consultant|Janssen: Honoraria|MSD: Advisor/Consultant|MSD: Speaker honoraria|Nordic pharma: Board Member|Nordic pharma: Honoraria|Pfizer: Advisor/Consultant|Pfizer: Honoraria|Shionogi: Advisor/Consultant|Shionogi: Honoraria|ViiV: Advisor/Consultant|ViiV: Honoraria **Nicola Petrosillo, n/a**, Becton & Dickinson,: Honoraria|MSD: Honoraria|ohnson & Johnson: Honoraria|Pfizer: Honoraria|Shionogi: Honoraria.

